# What change in body mass index is needed to improve metabolic health status in childhood obesity: protocol for a systematic review

**DOI:** 10.1186/s13643-016-0299-0

**Published:** 2016-07-26

**Authors:** Laura Birch, Rachel Perry, Chris Penfold, Rhona Beynon, Julian Hamilton-Shield

**Affiliations:** NIHR Biomedical Research Unit in Nutrition, Diet and Lifestyle, Level 3 University Hospitals Bristol Education Centre, Upper Maudlin Street, Bristol, BS2 8AE UK

**Keywords:** Obesity, Childhood, Adolescence, Body mass index, Metabolic health, Lifestyle intervention

## Abstract

**Background:**

Childhood obesity is one of the most serious, global, public health challenges and has adverse health consequences in both the short-and long-term. The purpose of this study is to establish the change in body mass index (BMI) needed to achieve improvements in metabolic health status in obese children and adolescents attending lifestyle treatment interventions.

**Methods:**

The following electronic databases will be searched from their inception: AMED, Embase, MEDLINE via OVID, Web of Science and CENTRAL via Cochrane library. Randomised controlled trials (RCTs) or cohort studies of lifestyle interventions (i.e. dietary, physical activity and/or behavioural therapy) for treating obesity in children and adolescents (4–18 years) will be included. Interventions that last less than 2 weeks and trials that include overweight participants or those with a secondary or syndromic cause of obesity will not be included. No language restrictions will be applied. Titles and abstracts will be assessed for eligibility by two reviewers, and data from full-text articles will be extracted using a standardised data extraction template. Reference lists of all included articles will be hand-searched for additional publications. A narrative synthesis of the findings will be presented, and meta-analysis will be conducted if considered appropriate.

**Discussion:**

This will be the first systematic review of studies to establish the change in BMI required to improve metabolic health status in obese children and adolescents.

**Systematic review registration:**

PROSPERO CRD42016025317

**Electronic supplementary material:**

The online version of this article (doi:10.1186/s13643-016-0299-0) contains supplementary material, which is available to authorised users.

## Background

Childhood obesity is one of the most serious global public health challenges of the twenty-first century [1]. In England, the latest figures from the National Child Measurement Programme, which measures the height and weight of around one million school children every year, showed that 9.1 % of children aged 4–5 years and 19.1 % of those aged 10–11 years were obese [2].

Childhood obesity has adverse health consequences in both the short-and long-term. Obese children and adolescents are at increased risk of developing metabolic disturbances, including hypertension, dyslipidaemia and insulin resistance, and they are more likely to become obese adults [3]. The presence of adverse changes in cardiac and vascular function and type 2 diabetes, previously considered adult morbidities, now being identified in obese children and adolescents [4–10] illustrates the urgent need for effective weight management treatment interventions to improve the metabolic health status of the paediatric population.

Moderate weight loss has been shown to have a positive impact on a large number of metabolic and cardiovascular risk factors [11, 12]. Evidence suggests that weight loss of 10 % in adults is often associated with marked clinical improvement in co-morbidities and therefore minimum weight management targets can be set to improve metabolic health in the adult population [13].

However, there is very limited evidence in relation to the amount of weight loss needed to effect beneficial change in metabolic health in the paediatric population. Paediatric weight management guidelines exist in many countries to promote best practice, but at present, many of these recommendations are based on low-grade scientific evidence [14]. Some studies have reported improvements in parameters of metabolic health as a function of improvements in measures of adiposity in children and young people [15–17], but there is yet to be a systematic quantification of the reduction in body mass or adiposity needed to achieve these improvements.

Given the scale of the obesity problem and the significant and sustained adverse effects on health, clinically effective treatment options are urgently needed. A Cochrane review of interventions available for treating obesity children concluded that combined dietary, physical activity and behavioural interventions can produce a significant and clinically relevant reduction in overweight in children and adolescents compared to standard care [14], but whilst most interventions describe statistically significant reductions in adiposity, few report on clinical change in metabolic health. This systematic review aims to establish the minimum change in body mass index (BMI) needed to improve metabolic health status in obese children and adolescents to inform clinical guidelines for paediatric weight management interventions and to guide outcome measures in future clinical trials.

## Objective

The objective of this study is to establish the minimum change in BMI needed to achieve improvements in markers of metabolic health in obese children and adolescents attending lifestyle treatment interventions.

## Methods

This protocol follows the Preferred Reporting Items for Systematic Reviews and Meta-Analyses for Protocols (PRISMA-P) 2015 reporting guideline (see Additional file 1).

### Eligibility criteria

Studies will be selected for inclusion in this review according to the following criteria:

#### Study design

Completed, published, randomised controlled trials (RCTs) and cohort studies, with or without follow-up periods.

#### Participants

Studies involving participants aged 4–18 years with a diagnosis of obesity using defined BMI thresholds. These thresholds include, but are not limited to, the 98th centile on the UK 1990 growth reference chart [18], 95th percentile on the US Centre for Disease Control and Prevention growth chart [19], the International Obesity Taskforce BMI for age cut-points [20] and the World Health Organisation growth references [21, 22], in addition to country-specific obesity thresholds using BMI reference data from their paediatric populations. Studies involving overweight individuals, pregnant females, the critically ill or those with endocrine disorders or syndromic obesity will not be included.

#### Interventions

Lifestyle treatment interventions that include dietary, physical activity and/or behavioural components with the objective of reducing obesity. No restrictions will be applied regarding the setting of the intervention. Interventions that last less than 2 weeks and those that involve surgical and/or pharmacological components (e.g. bariatric surgery, drug therapy) will not be included.

#### Outcomes

To be included, studies must report baseline and post intervention/change measurements of BMI/BMI standard deviation score (BMI-SDS or BMI-*Z*-score) and one or more of the following markers of metabolic health:

- Glucose
- Insulin sensitivity/resistance (homeostatic model assessment (HOMA))
- Lipid profile (triglycerides, total cholesterol, low-density lipoprotein (LDL)/high-density lipoprotein (HDL) cholesterol)
- Inflammation (C-reactive protein)
- Blood pressure (systolic, diastolic)
- Liver function
- Adiposity measures other than BMI including waist circumference and percentage body fat. Studies which report compatible alternative outcome measures (e.g. linear regression coefficients) will be considered.

#### Language

No restrictions will be applied regarding language. Non-English papers will be translated where possible.

### Information sources and search methods

Literature search strategies will be developed by an experienced systematic reviewer (RP) using a combination of Medical Subject Headings (MeSH) and keyword terms. The following electronic databases will be searched from their inception: AMED, Embase, MEDLINE via OVID, Web of Science and CENTRAL via Cochrane library. The search strategy for each database will be similar but revised appropriately for the specific database to take into account differences in controlled vocabulary and syntax rules. To ensure literature saturation, reference lists of all full-text articles will be hand-searched for additional original publications. Update searches will be conducted every 6 months to identify any studies published since the initial search. Conference abstracts will be used to help identify potential studies and authors will be contacted to establish if full-text articles are available.

### Study records

#### Data management

EndNote reference management software package will be used to manage all the search results throughout the review period. Literature search results will be imported into an EndNote library and duplicates will be removed.

#### Selection process

Titles and abstracts will be assessed for eligibility by two independent reviewers (LB, RP). Articles that appear to meet the inclusion criteria will be retrieved in full and independently considered for inclusion by two reviewers (LB, JHS). The reviewers will resolve disagreements in opinion of studies for inclusion through discussion, and the reasons for excluding studies will be recorded. Reference lists of included studies will be reviewed, and the full-text articles of any relevant studies identified will be retrieved and reviewed for inclusion by both reviewers.

#### Data collection process

Full-text articles for inclusion will be retrieved, and data will be extracted independently by two reviewers (LB, RP) using a standardised data extraction template. The template will be piloted by both reviewers before starting the review and modified as required to ensure consistency. Disagreements in opinion of data extracted will be resolved through discussion.

#### Data extraction

The following information will be extracted from each study:

- Study design (RCT/cohort)
- Definition of obesity
- Sample characteristics (size, inclusion/exclusion criteria, sex, age, pubertal status, ethnicity, socioeconomic status)
- Intervention (location, content, format, delivery)
- Metabolic health parameters measured (as described in the “Outcomes” section)
- Results
- Analysis methods
- Study limitations

#### Quality assessment

Full-text articles of all included research studies will be assessed for methodological quality by two independent reviewers (LB, RP) using the Quality Assessment form used in the 2004 *Health Technology Assessment* “Systematic review of the long-term effects and economic consequences of treatments for obesity and implications for health improvement” [23]. Any discrepancies between the two reviewers will be resolved through discussion or third-party adjudication.

As the focus of this review is the relationship between change in BMI and change in metabolic health parameters, rather than the specific interventions that effect these changes, more specific risk of bias tools (e.g. Cochrane Risk of Bias tool) were not considered appropriate for use in this review.

### Data synthesis

A narrative synthesis of the findings from the included studies will be presented, structured around the outcomes reported. Due to the diverse nature of the studies of interest, heterogeneity between studies is anticipated and therefore random effects models will be utilised to quantitatively synthesise all data where possible. Statistical heterogeneity will be assessed by visual inspection of forest plots and with the chi^2^ measurement. Since heterogeneity is difficult to assess when sample sizes are small, a cut-off of *P* < 0.10 for the chi^2^ measurement will be used to indicate heterogeneity. The *I*^2^ statistic will be used to measure variation in the effect size due to heterogeneity, and values greater than 50 % will be indicative of significant heterogeneity [24]. If studies have non-comparable intervention designs or outcome measures, we will pool effect sizes for comparable sub-sets of studies. Original authors will be contacted as required for any missing data. Where missing data remains, attempts will be made to estimate these missing values from other results reported in the studies or by imputing possible values using the mean of values from comparable studies. The likelihood of publication bias will be assessed through visual inspection of funnel plots and with Egger’s regression test, provided there are at least 10 studies included in the meta-analysis.

#### Sub-group analyses

Whether the minimum change in BMI necessary to effect change in metabolic outcomes differs between the following groups will be explored using sub-group analysis (provided there are sufficient studies in the sub-group) or meta-regression as appropriate: pubertal status, gender, RCTs vs observational studies.

#### Sensitivity analyses

To assess whether imputing missing data affects the pooled estimates, the main analyses will be repeated using a more conservative imputation method (i.e. using the maximum rather than mean appropriate value reported in other studies) and by excluding studies in which missing data was imputed.

## Discussion

This will be the first systematic review of lifestyle treatment interventions for obese children and adolescents to establish the minimum change in BMI required to improve metabolic health status. Whilst there is consensus that reducing adiposity in cases of obesity is beneficial, understanding how much body mass must be reduced by to positively affect metabolic health is important to ensure that weight management treatment interventions are appropriately designed and evaluated to achieve clinical rather than just statistical significance.

## Abbreviations

BMI, body mass index; BMI-SDS, BMI standard deviation score; HDL, high-density lipoprotein; HOMA, homeostatic model assessment; LDL, low-density lipoprotein; RCT, randomised controlled trial

## Additional file

Additional file 1: PRISMA-P (Preferred Reporting Items for Systematic Review and Meta-Analysis Protocols) 2015 checklist: recommended items to address in a systematic review protocol*. PRISMA is an evidence-based minimum set of items for reporting in systematic reviews and meta-analyses. (DOC 80.5 kb)
